# Central Nervous System Vasculitis: Still More Questions than Answers

**DOI:** 10.2174/157015911796557920

**Published:** 2011-09

**Authors:** Marco A Alba, Georgina Espígol-Frigolé, Sergio Prieto-González, Itziar Tavera-Bahillo, Ana García-Martínez, Montserrat Butjosa, José Hernández-Rodríguez, Maria C Cid

**Affiliations:** Vasculitis Research Unit, Department of Systemic Autoimmune Diseases, Hospital Clinic, University of Barcelona, Institut d´Investigacions Biomèdiques August Pi I Sunyer (IDIBAPS), Villarroel 170, 08036 Barcelona, Spain

**Keywords:** Vasculitis, Central nervous system.

## Abstract

The central nervous system (CNS) may be involved by a variety of inflammatory diseases of blood vessels. These include primary angiitis of the central nervous system (PACNS), a rare disorder specifically targeting the CNS vasculature, and the systemic vasculitides which may affect the CNS among other organs and systems. Both situations  are severe and convey a guarded prognosis. PACNS usually presents with headache and cognitive impairment. Focal symptoms are infrequent at disease onset but are common in more advanced stages. The diagnosis of PACNS is difficult because, although magnetic resonance imaging is almost invariably abnormal, findings are non specific. Angiography has limited sensitivity and specificity. Brain and leptomeningeal biopsy may provide a definitive diagnosis when disclosing blood vessel inflammation and are also useful to exclude other conditions presenting with similar findings. However, since lesions are segmental, a normal biopsy does not completely exclude PACNS. Secondary CNS involvement by systemic vasculitis occurs in less than one fifth of patients but may be devastating. A prompt recognition and aggressive treatment is crucial to avoid permanent damage and dysfunction. Glucocorticoids and cyclophosphamide are recommended for patients with PACNS and for patients with secondary CNS involvement by small-medium-sized systemic vasculitis. CNS involvement in large-vessel vasculitis is usually managed with high-dose glucocorticoids (giant-cell arteritis) or glucocorticoids and immunosuppressive agents (Takayasu’s disease). However, in large vessel vasculitis, where CNS symptoms are usually due to involvement of extracranial arteries (Takayasu’s disease) or proximal portions of intracranial arteries (giant-cell arteritis), revascularization procedures may also have an important role.

## 1. INTRODUCTION

The central nervous system (CNS) vasculature may be targeted by an heterogeneous group of inflammatory diseases. In its isolated, primary form, angiitis of the CNS (PACNS) is a rare form of vasculitis of unknown etiology primarily affecting small and medium sized vessels supplying the brain parenchyma, spinal cord and leptomeninges [1- 3]. PACNS results in signs and symptoms of CNS dysfunction with no clinically apparent participation of other organs. The CNS may also be targeted, among other territories, by systemic vasculitides [4, 5]. This review will focus on diagnostic and therapeutic aspects of PACNS and secondary CNS involvement by systemic vasculitides in adulthood. Primary and secondary CNS vasculitis in childhood have been addressed in excellent recent reviews [6-8].

## 2. PRIMARY CNS VASCULITIS

### 2.1. Epidemiology

Because of the rarity of PACNS and the absence of definitive diagnostic tests, epidemiologic studies are virtually inexistent. An annual incidence of 2.4 per million people has been recently estimated in North America [9]. PACNS has been reported in children [6-8] and in the elderly. However, it appears to be more frequent in males in their fourth and fifth decades of life [2, 9]. PACNS may represent 1.2% of vasculitis involving the CNS [3].

### 2.2. Pathogenesis

The pathogenesis of PACNS is unknown. Similar to other chronic inflammatory or autoimmune diseases, PACNS is thought to be triggered by infection. Cytomegalovirus, Ebstein-Barr virus, varicella-zoster virus, human immuno-deficiency virus, mycoplasma and chlamydia have been considered given the ability of these agents to produce vasculitic lesions [10-15]. However, in the majority of patients with PACNS a potential relationship with these or other infectious agents cannot be demonstrated. 

The granulomatous nature of the vascular inflammatory lesions in most cases suggests a Th1-mediated response [3, 16]. Th1-related cytokines may promote vascular inflammation in PACNS as suggested by several experimental models. Intracerebral injections of interferon-gamma have been shown to trigger inflammatory lesions and vasculitis in rats. [17]. Tumor necrosis factor (TNF) and interleukin-6 proinflammatory functions may also contribute to vascular inflammation in PACNS [18, 19]. TNF/TNF receptor p75 transgenic mice develop multifocal CNS ischemic injury secondary to vasculitis [18]. Elevated CSF IL-6 has been found in 3 patients with different types of vasculitis (polyarteritis nodosa, temporal arteritis and Behcet's disease) involving the CNS [19]. Current knowledge of the pathophysiology of PACNS is very limited delaying progress in the diagnosis and management of affected patients.

### 2.3. Pathology

**Fig. (1A).**
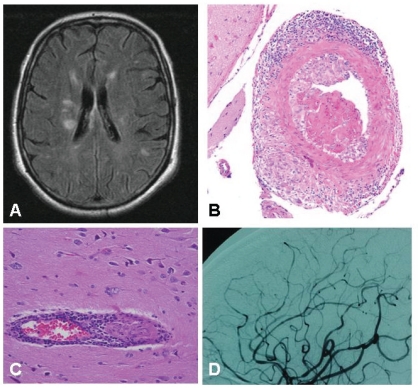
Multiple, non-specific, T2 hyperintense lesions in a 63- year old patient with suspected primary angiitis of the CNS who presented with headache and cognitive impairment. **B**) Granulomatous pattern of primary angiitis of the central nervous system. Transmural inflammation involves a muscular artery of the leptomeninges with prominent mononuclear (upper) and granulomatous (lower) adventitial inflammation as well as intimal injury with focal fibrin thrombus formation (hematoxylin and eosin 20×). Courtesy of Dr Carlo Salvarani. **C**) Inflammatory involvement of a small vessel. Courtesy of Dr Leonard H Calabrese. **D**) Multiple areas of irregular stenosis and ectasia in a 44year-old patient with biopsyproven PACNS. Courtesy of Dr Leonard H Calabrese.

PACNS typically involves small-medium sized arteries and veins, especially those located in leptomeninges and subcortical areas. The characteristic histopathologic findings consist of inflammatory infiltration of vessel walls by T lymphocytes and activated macrophages which undergo granulomatous differentiation with giant-cell formation [3, 16]. Inflammatory cells infiltrate the adventitia and subsequently progress through the artery wall causing fragmentation of the internal elastic lamina. Intimal proliferation and fibrosis leading to vascular occlusion is frequently observed [3, 16] (Fig. **1**). This granulomatous pattern is the most commonly seen and led to the previously used term granulomatous angiitis of the CNS [3, 16, 20]. However, granulomatous features may not be always observed and some specimens disclose the so-called atypical CNS angiitis patterns consisting in predominantly lymphocytic infiltrates (lymphocytic pattern), necrotizing vasculitis with fibrinoid necrosis (necrotizing pattern) or mixed patterns [20]. In some cases, B lymphocytes and plasma cells can also be observed [21]. Vascular β amyloid deposits may be found in a subset of patients [20]. 

Although most patients with PACNS present primarily with CNS dysfunction, necropsy studies may disclose clinically asymptomatic vasculitis in additional locations including lungs, kidneys and gastrointestinal tract [3, 5, 16]. Distinction from systemic vasculitis with prominent CNS involvement may be sometimes difficult to establish. 

### 2.4. Clinical Manifestations

Depending on the areas of the brain involved, PACNS may convey a wide variety of clinical findings. Moreover, disease severity and rapidity of progression may be highly variable among patients, increasing heterogeneity in clinical presentation.

In the largest series reported including 101 patients [9], the median age at diagnosis was 47 years (range 17-84 years). The majority of patients presented with subacute manifestations of diffuse CNS dysfunction. Acute presentation was highly unusual. The most common initial symptoms were headache (63%) and cognitive impairment (50%). Headaches were initially of low intensity and progressively worsened. Cognitive impairment was also insidious. Focal symptoms usually appeared later in the course of the disease and included hemiparesis (44%), stroke (40%), aphasia (28%), transient ischemic attack (28%), ataxia (19%), seizures (16%), dysarthria (15%) and blurred vision or decreased visual acuity (11%). Infrequent manifestations, occurring in less than 10% of patients, included intracranial hemorrhage, amnesic syndrome, spinal cord manifestations such as paraparesis o quadriparesis, parkinsonism, vertigo, dizziness or cranial nerve palsy. Most patients had multiple manifestations*.* Other published series report similar findings [22, 23]. 

In order to facilitate clinical recognition and early diagnosis, clinical manifestations have been grouped in three major phenotypes: 1) Acute or more commonly subacute encephalopathy, presenting as a confusional syndrome with progression to stupor and coma; 2) Disease presentation resembling atypical multiple sclerosis with a variety of focal symptoms such as optic neuropathy, brain stem episodes, seizures, headaches, encephalopathic episodes or hemispheric stroke-like events and 3) Intracranial mass lesions, with headache, drowsiness, focal signs and elevated intracranial pressure [24, 25]. 

 It has also been suggested that predominant involvement of small versus medium-sized vessel may influence disease presentation. Small-vessel PACNS manifests as a subacute or acute encephalopathy with persistent headaches, cognitive impairment, confusion, and seizures. MRI usually discloses marked meningeal contrast enhancement whereas angiography may not reveal changes because the affected vessels are small, beyond the detection threshold [26, 27]. This form of PACNS may respond to glucocorticoid monotherapy but 25% of patients relapse. In contrast, when medium-size vessels are involved, in addition to headaches and general CNS dysfunction, focal neurologic deficits and stroke are more common and angiography is more likely to reveal vascular abnormalities [9, 26, 27]. Four clinical features are associated with an increased mortality in patients with PACNS: focal neurological deficit, cognitive impairment, cerebral infarction and involvement of larger vessels [9]. 

General symptoms and findings suggesting some extent of systemic involvement may occur. Fever, weight loss, *livedo reticularis*, rash, peripheral neuropathy, arthritis and night sweats may be recorded in 20% of patients [2, 9]. 

### 2.5. Diagnosis

The diagnosis of PACNS is a challenge because of the lack of highly sensitive and specific diagnostic tests. Clinical, analytical, neuroimaging, and histopathologic data are important, both in supporting the diagnostic suspicion and in excluding other conditions which may present with similar features.

#### 2.5.1. Laboratory Test Abnormalities 

Routine laboratory tests are frequently within the normal range [2, 9, 28]. In some patients features of systemic inflammatory response including anemia, leukocytosis and moderately increased acute phase reactants (ESR, C-reactive protein and platelet counts) can be observed [2, 9]. Laboratory tests are useful to rule out other diseases which may present with similar symptoms such as infection, systemic vasculitis, malignancy, drug abuse and hypercoagulability states [5, 28, 29].

Cerebrospinal fluid (CSF) is abnormal in 80-90% of patients [9]. Increased protein concentration is the most common finding. In a series of 101 patients, mean CSF protein concentration was 7 gr/L (range 1.5-10.3 gr/L) [9]. Pressure is increased in 50% of patients and elevated lymphocyte counts may be observed in 50-80%. CSF oligoclonal immunoglobulins may be found in up to 50% of individuals with PACNS [5, 23]. CSF pleocytosis is modest, rarely exceeding 250 cells/μL. Higher leukocyte counts and the presence of neutrophils are uncommon and, when present, should alert for possible infection [2]. CSF analysis is useful to exclude infection and malignancy and appropriate bacterial and fungal stains, viral polymerase chain reactions, and flow cytometry studies should be performed.

#### 2.5.2. Imaging

##### 2.5.2.1. Magnetic Resonance Imaging (MRI) and Magnetic Resonance Angiography (MRA)

MRI is sensitive but not specific in revealing changes associated with PACNS [30]. Lesions are frequently multiple and bilateral and include parenchymal or meningeal enhancing areas, ischemic areas or infarcts in the cortex, deep white matter, or periventricular white matter (Fig. **1A**). It may also disclose hemorrhagic lesions [31, 32]. The sensitivity of MRI in biopsy-proven PACNS is very high, disclosing abnormalities in 97% of cases [22, 32-34] but abnormal findings are non specific. Diffusion weighted imaging is highly sensitive in detecting diffusion abnormalities and may be useful in patients with normal MRI [35]. MRA has limited sensitivity and is only able to disclose abnormalities in the largest intracranial vessels. The same limitations apply to CT-angiography [33, 34]. 

##### 2.5.2.2. Conventional Angiography

Conventional angiography is the most specific imaging technique for the diagnosis of PACNS and, compared to MRA is able to detect abnormalities in smaller vessels. Typical angiographic features of PACNS include multiple “beading” or segmental narrowing in large, intermediate, or small arteries with interposed regions of ectasia or normal luminal architecture [31-33] (Fig. **1D**). Beading may be smooth or irregular and typically occurs bilaterally. Additional changes include aneurysms, collateral flow, isolated areas of vessel narrowing in multiple branches, circumferential or eccentric vessel irregularities, multiple occlusions with sharp cutoffs, and apparently avascular mass lesions [31-33]. 

Although findings from CNS conventional angiograms may support the diagnosis of PACNS and can be used to direct the site of biopsy, none of these findings alone is diagnostic because similar images can be present in other diseases (Tables **1** and **2**) [2, 5, 22, 28, 36-38]. 

Although essential for diagnosis, angiography has limited sensitivity and specificity. Patients with biopsy-proven PACNS may have normal appearing angiograms and, conversely, biopsies of angiographically abnormal vessels have been reported as normal [2, 5, 28]. The sensitivity of angiography in detecting PACNS ranges from 20% to 90% [1, 9, 31, 35, 37, 38] and specificity from 20 to 60% [1, 9, 31, 34]. The sensitivity of cerebral angiography decreases along with the caliber of the involved vessels, being most sensitive for involvement of large-medium sized vessels. Angiography is not exempt of side effects. About 0.8% of patients subjected to angiography experience additional neurologic deficits as an adverse event related to the procedure [32]. However, given the severity of PACNS and the difficulties in achieving an accurate diagnosis, the risk/ benefit is acceptable and conventional angiography is recommended as a key diagnostic procedure. 

##### 2.5.2.3. Histopathologic Examination 

Brain biopsy is considered the gold standard for the diagnosis of PACNS but reveals diagnostic histopathologic abnormalities in only 50% to 75% of cases [1] (Fig. **1B** and **C**). The role of brain biopsy in PACNS is not limited to proving inflammation of blood vessels: it is also important to excluding other conditions such as infection, malignancy, or degenerative diseases for which completely different treatment approaches are required (Table **1**) [5, 27]. 

In the largest series of PACNS patients undergoing surgical biopsy, including 43 patients, diagnostic sensitivity of brain biopsy was 63% [20]. In this series, the distribution of the various morphologic patterns was as follows: acute necrotizing (14%), purely lymphocytic (28%) and granulomatous (58%), with no statistically significant differences in disease aggressiveness or response to treatment among them. Interestingly, 78% of the biopsies directed to an imaging abnormality were diagnostic, whereas none of the blind biopsies demonstrated vasculitis. Biopsies including leptomeninges were slightly more sensitive in detecting vasculitis than those not including it (58% vs. 40%). In accordance with these results other authors have reported a sensitivity of brain biopsy around 50% [2, 16]. The high proportion of negative biopsies in patients with clinical and radiographic features highly suggestive of PACNS may be explained by the segmental nature of lesions. Moreover biopsies are usually taken from the superficial parenchyma and leptomeninges and, in some instances, involved vessels are of greater size and are located deeper from these areas [20]. To maximize the diagnostic sensitivity of the procedure it is recommended that biopsies are performed in abnormal areas detected by previous imaging and include leptomeninges. Stereotactic biopsy is recommended for mass lesions only [20, 25].

Occasionally, amyloid deposits can be observed [20, 25]. These are more frequently found in samples with a granulomatous pattern and those presenting as mass lesions [20, 25]. Clinically, patients with amyloid deposits are older and more frequently presenting with acute onset and cognitive impairment [39]. Clinical outcome and response to treatment seems to be similar to that of patients with no amyloid deposits [39].

##### 2.5.2.4. Diagnostic Criteria

**Fig. (2).**
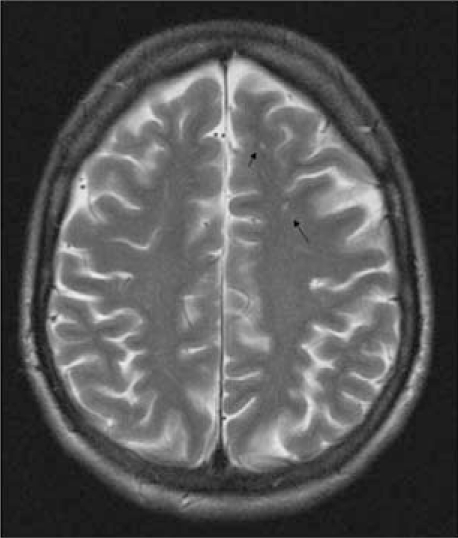
Puntiform T2 hyperintense white matter lesions in a 40- year old woman with Susac’s syndrome. This patient also had sensorineural hypoacusia and bilateral retinal artery branch occlusions as part of the syndrome.

Since histopathologic confirmation of PACNS is not always feasible, Calabrese and Mallek proposed a series of diagnostic criteria combining, clinical, imaging and histopathologic findings [1]. These include: 1) neurologic deficit that remains unexplained after a vigorous diagnostic workup, including lumbar puncture and neuroimaging studies, 2) angiographic abnormalities highly suggestive of vasculitis or histopathologic evidence of vasculitis within the CNS and 3) no evidence of systemic vasculitis or any other condition to which the angiographic or pathologic findings can be attributed. These conditions are listed in Table **1** (Fig. **2**).

##### 2.5.2.5. Treatment

No randomized controlled trials or prospective studies have been performed with patients with PACNS. Therefore, therapeutic recommendations are based on extrapolation of data obtained from trials performed in other severe systemic vasculitides, retrospective studies, small case series and expert opinion [2, 5, 40]. In a retrospective review of treatments received by 101 patients diagnosed with PACNS (70 by angiography, 31 by biopsy) Salvarani *et al.* found that 97 patients were treated with glucocorticosteroids, 25 of them with 1gr intravenous methyl-prednisolone pulses and the remaining with oral prednisone at a median dose of 60 mg/day [9]. Forty-nine patients received an immunosuppressive agent: 46 cyclophosphamide (oral at 150 mg/day or intravenous at around 1 gr/month) and 3 azathioprine. A favorable response was observed in 81% of the patients treated with glucocorticoids alone and in 81% of those receiving both prednisone and cyclophosphamide. Given the retrospective nature of the survey it is not possible to conclude that immunosuppressive agents are not necessary since the group receiving cyclophosphamide may have been considered more severe by treating physicians.

Treatment with glucocorticoids (oral prednisone or equivalent at 60 mg/day preceded by three 1 gr intravenous pulses in severe cases) should, then, be started as soon as CNS vasculitis (primary or secondary) is clinically suspected and infectious diseases reasonably excluded. Prednisone can be quickly tapered if the diagnosis is eventually ruled out. When the diagnosis of CNS vasculitis is also supported by angiography or biopsy and mimics are convincingly excluded, cyclophosphamide (oral at 150 mg/day or 1gr monthly pulse) is recommended. Pulse intravenous cyclophosphamide has equivalent efficacy in inducing remission but it is less toxic than daily oral cyclophosphamide in systemic vasculitis [40]. By analogy to severe systemic vasculitis, switch to a safer immunosuppressive agent (azathioprine, methotrexate or mycophenolate) may be considered after 4-6 months of cyclophosphamide treatment [40-43]. All patients should be given calcium and vitamin D, bone protection agents and *Pneumocystis* infection prophylaxis [5].

Recently it has been shown that rituximab is equally effective than cyclophosphamide in inducing remission in severe ANCA-associated systemic vasculitis [44, 45]. Rituximab has also been successful in treating SLE patients with CNS involvement [46], but there is no experience with rituximab in PACNS. Two glucocorticoid and cyclophosphamide refractory cases responding to TNF blockade have been reported [47].

Immunossuppressive treatment should be maintained for 2-3 years [2, 5]. It is important to keep in mind that about 25% of patients may relapse [9]. Response to treatment must be monitored by periodic neurologic evaluation and serial MRI examination every 3-4 months [2, 28].

## 3. REVERSIBLE CEREBRAL VASOCONSTRICTION SYNDROME (RCSV)

RCVS is a recently proposed term to describe the physiopathologic substrate of a group of conditions characterized by prolonged but reversible vasoconstriction of the cerebral arteries [48]. Previously, these syndromes were referred as benign angiopathy of the central nervous system and, for many years, there has not been a clear distinction between RCVS and true primary angiitis of the CNS. RCVS has received a variety of names: Call-Fleming syndrome, thunderclap headache with reversible vasospasm, migrainous vasospasm or migraine angiitis, postpartum angiopathy, or drug-induced cerebral arteritis or angiopathy [48]. 

RCVS may occur spontaneously but in most instances is associated with precipitating factors including the use of vasoactive substances (i.e. ergotamine derivatives, amphetamines and nasal decongestants) other drugs (i.e selective serotonin-reuptake inhibitors, contraceptives), recreational drugs (cannabis, ecstasy, LSD, cocaine, alcohol), late pregnancy or puerperium, sexual intercourse, and catecholamine producing tumors [48-50]. The most characteristic initial clinical manifestation include hyperacute severe and recurrent headache that can be associated with neurologic symptoms and signs [48]. Headache is usually diffuse although may be also localized, preferentially in the occipital area, and may be associated with nausea, vomiting and photosensitivity. Other clinical manifestations include visual dysfunction, transient ischemic attacks and seizures [48]. The major complication of RCVS is stroke that can eventually lead to permanent sequelae and even death [48, 49]. Although the pathophysiology of RCVS is not known, the prevailing hypothesis considers that there is a transient disturbance in the control of cerebral vascular tone [48].

In the largest series reported including 67 patients [49], there was a female predominance (67%) with a mean age at diagnosis of 42.5±11.8 years (range 19-70 years). Precipitating factors were identified in 63%, being the use of vasoactive substances the most frequent (55%). The presenting symptom in all cases was recent severe headache, and this was the only symptom in 76%. Among the 67 patients, 94% had multiple thunderclap headaches (mean of 4.5 episodes) that recurred over a mean period of 1 week. In this series, early complications (within the first week) included cortical subarachnoid hemorrhage (22%), reversible posterior leukoencephalopathy (9%), intrecerebral bleeding (6%) and seizures (3%). Delayed complications (after the first week) included transient ischemic attack in 16% and cerebral infarcts in 4%. The overall outcome in this series was good, with no relapses during a 16±12.4 month follow-up period and only 4% of patients had persistent neurological deficits. 

In the absence of validated diagnostic criteria, Calabrese *et al.* [48] proposed a set of key elements required for the diagnosis of RCVS. These include severe, acute headaches, with or without additional neurologic signs or symptoms, normal or near to normal cerebrospinal fluid analysis, neuroimaging tests (transfemoral angiography, CT angiography or MRA) documenting multifocal segmental cerebral artery vasoconstriction, with no evidence for aneurysmal subarachnoid hemorrhage, and reversibility of angiographic abnormalities within 12 weeks [47-49]. Treatment usually consists of calcium-channel blockers [48-51] and brief glucocorticoid courses [50, 52]. 

The distinction of PACNS and RVCS is important because of the different prognosis and treatment requirements. Key elements for distinction have been proposed [2, 48] and are summarized in Table **2**. PACNS typically affects middle-aged men whereas RVCS is primarily a disease of women between 20-40 years. In the latter almost 60% of patients report a precipitating event [48], usually exposure to vasoactive substances. Headache in PACNS is indolent and progressive [9] whereas headache in RVCS is acute and severe [2, 48, 49]. Unless complicated by bleeding or infarct, MRI does not disclose major changes in RVCS whereas MRI is abnormal in 97% of cases with PACNS [9, 50]. By definition, angiographic abnormalities substantially or completely reverse within approximately 3 months. 

## 4. SYSTEMIC VASCULITIDES INVOLVING THE CNS

The CNS vasculature can be targeted by systemic vasculitis (Table **3**). Usually CNS involvement coexists with other clearly apparent systemic manifestations but some patients may present primarily with prominent symptoms of CNS dysfunction [4, 5, 53]. In systemic vasculitis targeting small-medium sized vessels, CNS involvement is a predictor of poor/guarded prognosis [54, 55] and is one of the factors considered to recommend aggressive treatment with cyclophosphamide in addition to high-dose steroids [40, 54, 55]. However, in large-vessel vasculitis, CNS involvement may benefit from vascular intervention procedures (angioplasty, derivative surgery), antiplatelet or anticoagulation treatment in addition to high dose glucocorticoids rather than intensification of immunossupressive therapy [56-58].

### 4.1. CNS Involvement by Small and Medium Sized Vessel Vasculitis

Globally, cerebrospinal involvement is infrequent in small-medium size vessel vasculitis, including Wegener’s granulomatosis, microscopic polyangiitis, Churg-Strauss syndrome, polyarteritis nodosa, cryoglobulinemic vasculitis, and Behçet’s disease. CNS involvement occurs in less than 15% of patients in most series.

#### 4.1.1. Wegener Granulomatosis (WG)

The prevalence of CNS manifestations in WG ranges from 2.7% to 9% in large series of patients [59-61]. Neurological involvement may account through 3 major mechanisms: vasculitis involving CNS vessels, granulomatous lesions located in the brain, meninges or cranial nerves and direct extension of destructive granulomatous tissue from nasal or paranasal structures [59-62].

Cerebral vasculitis is the most frequent CNS lesion and may present with headache, visual disturbances, seizures, confusion, ischemic stroke, intracerebral or subarachnoid haemorrhage, venous thrombosis or dementia [62, 63]. Granulomatous inflammation and thickening of the duramater, pachymeningitis, may present with chronic headache, multiple cranial nerve palsies, seizures, meningeal signs, encephalopathy, proptosis, limb palsy or ataxia [62-65]. Pituitary involvement leads to central diabetes insipidus, panhypopituitarism or a combination of hormone deficiencies [66]. In these patients, MRI is the image technique of choice because it can reveal ischemic or hemorrhagic lesions, dural thickening, pituitary involvement or enhancement of inflamed orbital and paranasal mucosa [63]. In the case of dural involvement, tissue biopsy may disclose granulomatous pachymeningitis [66]. 

#### 4.1.2. Microscopic Polyangiitis (MPA)

In a series of 85 patients, CNS involvement was present in 10 cases (11.8%) and CNS vasculitis was the cause of death of one of them [67].

There are only scattered case reports of CNS manifestations related to MPA in the literature. Multiple bilateral cerebral infarctions [68], multiple hemorrhagic infarction of the cerebral cortex caused by CNS vasculitis [69], capsular warning syndrome and subsequent stroke [70] and pachymeningitis have been occasionally reported [71, 72].

#### 4.1.3. Churg-Strauss Syndrome (CSS)

In the largest published series of CSS patients the CNS is reported to be involved in 8% to 14% of patients [73-77]. 

**Fig. (3A).**
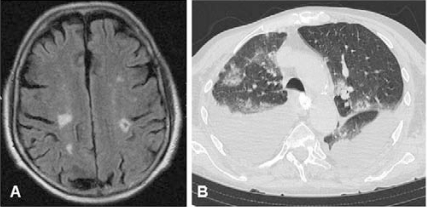
Multiple brain infarcts in a patient with Churg-Strauss syndrome. **B**) CT scan from the same patient disclosing pulmonary infiltrates and bilateral pleural effusion. Toracocentesis disclosed predominance of eosinophils in pleural fluid exudate.

Cerebral infarction is the most frequently reported manifestation of CNS involvement [75, 77], probably as result of cerebral vasculitis (Fig. **3**). Additional less commonly reported CNS events include intracerebral haemorrhage [78, 79] and pachymeningitis [80, 81].

#### 4.1.4. Polyarteritis Nodosa (PAN)

In a recent series of 348 patients diagnosed with PAN over a 42-year period, 4.6% presented with central nervous system-related abnormalities [82]. Earlier studies reported a higher prevalence, between 15 and 65% [83]. Perhaps in present days, earlier recognition of the disease with prompt treatment prevents development of severe complications. It is important to remark that, widespread ANCA and cryoglobulin testing has led to re-classification of a substantial proportion of patients with necrotizing vasculitis previously diagnosed with PAN, which, in fact, has become a much more infrequent disease [84]. 

**Fig. (4A).**
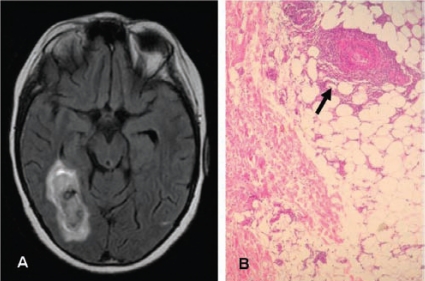
Hemorrhagic brain infarct in a patient with systemic poyarteritis nodosa. This patient also had hypertension, postprandial abdominal pain, multineuritis and livedo reticularis. **B**) Skin biopsy of the same patient disclosing necrotizing arteritis in the subcutaneous tissue.

 In an extensive literature review, three major clinical presentations related to CNS involvement have been recognized in PAN: 1) diffuse encephalopathy characterized by cognitive impairment, disorientation or psychosis (8% to 20%), 2) seizures (focal or generalized) and 3) focal neurologic deficits [83]. Accelerated hypertension may also contribute to diffuse encephalopathy in some patients [83]. Abnormal findings reported in neuroimaging studies (MRI and CTscan) include cerebral infarctions located in the brain (cortical or subcortical), cerebellum or brainstem and cerebral hemorrhages [85, 86] (Fig. **4**). 

#### 4.1.5. Cryoglobulinemia

CNS involvement is uncommon in cryoglobulinemic vasculitis. In a retrospective series of 209 patients [87], CNS involvement was detected in 3. In a prospective study of 40 patients with mixed type II cryoglobulinemia vasculitis [88] specifically investigating signs of CNS dysfunction, 89% of the patients had some cognitive impairment, being attention the aspect most commonly altered (70.3%), followed by alterations in executive functions and visual construction. Whether these abnormalities are due to CNS vasculitis, co-morbidities, glucocorticoid, immunosuppressive or antiviral treatments or a combination of factors is unclear.

Clinical features of CNS involvement in cryoglobulinemia include encephalopathy, stroke, transient ischemic attacks, lacunar infarctions and hemorrhage [89, 90]. Most of the cases reported are associated to hepatitis C virus infection.

#### 4.1.6. Behçet’s Disease

The frequency of neurological involvement in Behçet’s disease ranges from 5.3% to 14.3% in prospective studies [91, 92]. Neuro-Behçet occurs more frequently in patients aged 20 to 40 years and is 2-8 times more frequent in men than in women. Neurological manifestations commonly appear when other systemic features are present. CNS involvement is the first disease manifestation in less than 6% of patients with neuro-Behçet [93]. CNS involvement in Behçet’s disease may occur through 2 major mechanisms: meningoencephalitis and vascular disease. 

Meningoencephalitis is usually subacute and predominantly involves the brainstem but may extend to basal ganglia, thalamus, cortex and white matter [93, 94]. The spinal cord and cranial nerves may also be affected. In the largest series of patients with neuro-Behçet [92] the most common clinical symptoms were pyramidal signs (96%), hemiparesis (60%), behavioural changes, headache and sphincter disturbance or impotence. Less common manifestations were paraparesis, meningeal signs, movement disorders, brainstem signs, seizures, hemianopsia, aphasia, psyachiatric disturbances or cerebellar syndrome. CSF analysis was abnormal 70–80% disclosing moderately elevated protein concentration and pleocytosis with neutrophilia at early stages [89]. MRI discloses hyperintense T2 lesions with contrast enhancement and edema. Lesions are usually unilateral and are located in the upper brainstem extending towards the thalamus and basal ganglia [95]. Tumor-like lesions may occasionally occur [93].

The most common manifestation of vascular neuro-Behçet is central venous thrombosis with signs and symptoms of intracranial hypertension, including papilledema. Intracranial aneurysms and ischemic stroke may also occur but are infrequent complications. Combined parenchymal and vascular involvement may be seen in 20% of patients with neuro-Behçet [93]. Patients with neuro-Behçet are treated with high-dose glucocorticoids and cyclophosphamide. Blocking TNFα with infliximab may be useful in refractory patients.

### 4.2. Large Vessel Vasculitis

Both giant-cell arteritis of the elderly and Takayasu disease may convey CNS involvement.

#### 4.2.1. Giant Cell Arteritis

GCA preferentially targets the cranial vessels. Consequently the most common ischemic complications occur in territories supplied by the carotid and vertebral arteries. Although GCA is considered a large to medium sized vessel vasculitis, small cranial vessels are frequently affected [96] and the most frequent ischemic complication, visual loss, derives from involvement of the small arteries supplying the optic nerve [97-100]. Visual loss occurs in 15-20% of patients [97-100]. In 80-90% of cases visual impairment is due to anterior ischemic optic neuritis secondary to involvement of the posterior cilliary arteries supplying the optic nerve [101, 102]. Occlusion of the retinal artery is less frequent and underlies visual loss in 10% of cases [99, 100]. 

**Fig. (5A).**
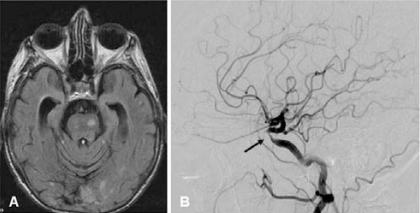
Multiple infarcts in the cerebral pons, cerebellum, and occipital lobes in a patient with biopsy-proven giant-cell arteritis who developed ataxia and cognitive impairment after the initiation of glucocorticoid therapy. **B**) Cerebral angiography displaying carotid siphon stenosis in a patient with biopsy-proven giant-cell arteritis who developed recurrent transient ischemic attacks (aphasia and hemiparesis) in spite of high-dose glucocorticoids, antiplatelet and anticoagulant therapy. This lesion was successfully treated with percutaneous transluminal angioplasty (57).

Ischemic stroke or multiinfarct dementia occurs in 3-6% of patients and is due to inflammatory involvement of the intracranial branches of the carotid and vertebral arteries. [97, 100, 103, 104]. When explored, ultrasonography of the supraaortic branches are frequently normal [103, 104]. Usually, inflammation is limited to the most proximal, extradural part of these arteries. In some series, strokes are more frequent in the vertebrobasilar territories contrarily to atherosclerotic occlusions which are more frequent in the carotid branches [103]. Brain infarcts are frequently multiple, indicating involvement of various branches, reduced flow from proximal stenosis, distant embolization of proximal thrombi, or a combination of these [97, 103, 104] (Fig. **5A**). Although thrombosis is uncommonly seen in temporal artery biopsies, necropsy studies from patients dying from GCA-related stroke, frequently disclose thrombosis as a precipitating event [100]. Mortality of GCA-related stroke is about 30% [103, 104]. 

Stroke is more frequent among individuals with visual loss indicating that some individuals may be more prone to develop intracranial involvement and related complications [97, 98]. Several studies indicate that individuals with prominent extracranial large- vessel involvement are less prone to develop cranial ischemic complications, suggesting heterogeneity in patterns of vascular targeting by GCA [105- 107]. Several studies indicate that traditional vascular risk factors are more frequent and the systemic inflammatory response is weaker in patients with GCA-related ophthalmic and neurologic ischemic complications, making early diagnosis and follow up more difficult [97-100, 108]. High dose glucocorticoids usually prevent progression of visual impairment. Intravenous methylprednisolone pulses are usually administered in this setting but there is no proof that this approach is more effective than the standard daily 60 mg dose. In 10-27% of patients presenting with visual symptoms, vision may continue to deteriorate during the first 1-2 weeks after the beginning of glucocorticoid treatment [99]. Antiplatelet or anticoagulant therapy is usually given in these circumstances with variable results [99, 101, 102]. After this initial period, the risk of developing subsequent disease-related visual loss is low, about 1% in 5 years [109].

Stroke frequently occurs during the first weeks after the initiation of glucocorticoid treatment. Besides adding antiaggregants, anticoagulants, or both, the classical approach to this situation has been intensifying glucocorticoid and immunosuppressive therapy. However, a recent report indicate that some patients with proximal lesions may better benefit from intracerebral percutaneous angioplasty [57] (Fig **5B**).

#### 4.2.2. Takayasu Arteritis

**Fig. (6).**
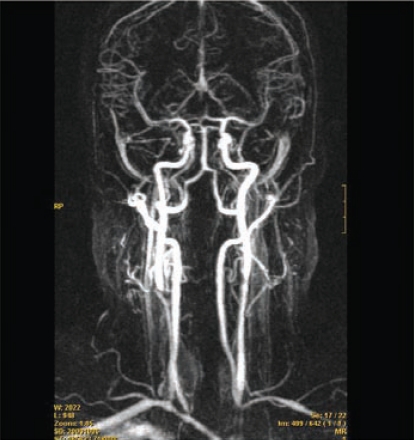
Multiple stenoses in the carotid and vertebral arteries in a 38-year old patient with Takayasu disease complaining from lightheadedness and slight dizziness.

Non specific neurologic manifestations such as headache, dizziness of variable intensity, and lightheadedness are highly frequent in patients with Takayasu’s arteritis, occurring in 57-90% in most series [58, 110, 111] (Fig. **6**). More severe complications include visual disturbances or visual loss, syncope, transient ischemic attacks and stroke. Most of these symptoms/complications can be related to extracranial steno-occlusive lesions in the subclavian (with subsequent arm-steal syndrome), carotid and vertebral arteries which results in decrease brain flow [112, 113]. Stroke occurs in less than 10% of cases in large cohorts but it is among the leading causes of premature death in these patients [58]. Strokes are usually ischemic and secondary thrombosis of stenotic vessels with subsequent embolization may be precipitating events. It is important to remark that cardiomyopathy secondary to aortic valve insufficiency due to aortic root dilatation or hypertension occurs in about 10% of patients with Takayasu’s disease and may also result in thromboembolic strokes [58]. Hemorrhagic stroke related to hypertension has also been reported [112].

Intracranial artery involvement seems to be uncommon. A recent prospective study using ultrasonography and MRI in 17 patients with neurologic symptoms, disclosed signs of intracranial involvement in 7 patients [113]. However, no angiography was performed and it was not possible to discern whether these findings were related to vasculitis or previous embolization. Autopsy studies including the brain are scarce in Takayasu’s disease, but intracranial involvement seems to be unusual. However, at least one patient with vasculitis of intracranial arteries has been reported [114].

Glucocorticoids and in most instances immunossupressive agents are mandatory to induce and maintain remission in patients with Takayasu disease. Cyclophosphamide and methotrexate have been useful in open-label studies and mycophenolate has been also tried in small case series [58, 110, 115]. Because of its side effects cyclophosphamide is usually avoided and other immunosuppressive agents are preferred, since Takayasu disease is a relapsing condition usually targeting young women [58, 110, 115]. TNF blockade has provided benefit to patients refractory to other therapies [116]. Angioplasty, stenting and by-pass surgery are very important in the management of severe neurological involvement [56, 58]. For better results, revascularization procedures, should be avoided, when possible, during periods of active disease and be performed to patients in remission [115].

## CONCLUSIONS

CNS vasculitis, either primary or complicating systemic vasculitis is uncommon. However CNS involvement is a major determinant of severity, morbidity and mortality in patients with vasculitis. Diagnosis of PACNS is a challenge and requires high index of clinical suspicion. Diagnosis is supported by neuroimaging and histologic data but requires exclusion of other conditions with the appropriate work-up. Neuroimaging techniques are pivotal not only to support the diagnosis but also in the follow up of affected patients. PACNS or CNS involvement by systemic vasculitis requires prompt recognition and aggressive treatment in order to reduce mortality and preserve function.

## Figures and Tables

**Table 1.** Mimics of Primary Angiitis of the Central Nervous System

**INFECTIOUS VASCULITIS**
Viral (HIV, varicella zoster, progressive multifocal leukoencephalopathy)
Borreliosis
Tuberculosis
Syphilis
Whipple’s disease
Endocarditis

**INFLAMMATORY DISEASES**
Systemic Vasculitis
Behçet’s disease
Neurosarcoidosis
Systemic lupus erythematosus

**NON-INFLAMMATORY VASCULOPATHIES**
Reversible vasoconstriction syndromes (RVCS)
Atherosclerosis
Susac’s syndrome
Radiation vasculopathy
Ehlers-Danlos disease
Kohlmeyer- Degos disease
Fibromuscular dysplasia
Fabry’s disease
Moya-moya disease
Amyloid angiopathy
CADASIL
*Pseudoxanthoma elasticum*
Mitochondrial diseases (MELAS)

**DEMYELINATING DISEASES**
Multiple sclerosis
Acute disseminated encephalomyelitis

**THROMBOEMBOLIC DISEASES**
Antiphospholipid syndrome
Hypercoagulability states
Cholesterol embolisms
Cardiac myxoma
Nonbacterial thrombotic endocarditis

**MALIGNANCIES**
Multifocal glioma
CNS lymphoma
Angiocentric lymphoma
Intravascular lymphoma (malignant angioendotheliomatosis)

**Table 2.** Clinical, Laboratory, Imaging and Histopathologic Characteristics Useful to Distinguish RVCS from PACNS

	RVCS	PACNS

**Clinical data**		
age	20-40 years	40-60 years
gender	primarily women	more frequent in men
trigger (drugs, postpartum etc)	frequently identified	absent
headache	acute and severe	insidious
cognitive impairment	unusual	frequent

**CSF**	Normal or minimal protein increase	Abnormal (increased protein concentration and mild pleocytosis)

**MRI**	Normal (>70%) *	Abnormal in 90%. Small infarcts in grey and white matter in multiple vascular territories, diffuse white matter lesions, mass lesions

**Angiography**	Abnormal:diffuse areas of multiple stenoses and dilatations **	May be normalSingle or multiple abnormalities(cut-offs, lumen irregularities, avascular mass lesion)

**CNS / leptomeningeal biopsy**	Normal	Vasculitis

* Except when complicated by stroke, intraparenchymal or cortical subarachnoid hemorrhage or posterior reversible leukoencephalopathy.

** Angiographic abnormalities are required for diagnosis but must be reversible in 6-12 weeks.

**Table 3.** Primary Systemic Vasculitis Most Frequently Involving the CNS in Adults

SMALL-MEDIUM VESSEL VASCULITIS(*)
Wegener’s granulomatosis
Microscopic polyangiitis
Churg-Strauss syndrome
Cryoglobulinemic
vasculitis
Behçet’s disease

MEDIUM VESSEL VASCULITIS (**)
Polyarteritis nodosa

**LARGE-VESSEL VASCULITIS**
Giant-cell arteritis
Takayasu’s arteritis(***)

In children, Henoch-Shönlein purpura

* and Kawasaki disease

** may occasionally involve the CNS

*** Neurologic complications in Takayasu’s arteritis are mainly due to involvement of extacranial vessels.
